# Impact of ultrasound-guided fine needle aspiration cytology for diagnosis of thyroid nodules

**DOI:** 10.1097/MD.0000000000017192

**Published:** 2019-09-20

**Authors:** Jing Wang, Jing Liu, Zhe Liu

**Affiliations:** a Department of Endocrine and Metabolism; b Department of Laboratory, The First Hospital of Yulin, Yulin; c Department of Cardiology, Yanan University Affiliated Hospital, Baota District, Yan’an, China.

**Keywords:** sensitivity, specificity, thyroid nodule, ultrasound-guided fine needle aspiration cytology

## Abstract

**Background:**

Previous clinical studies have reported that ultrasound-guided fine needle aspiration cytology (UGFNAC) can be used for the diagnosis of thyroid nodules (TN) effectively. However, no study has systematically explored its diagnosis accuracy in patients with TN. Thus, this study will assess its diagnosis accuracy for TN.

**Methods:**

We will perform a comprehensive literature search from the following databases from their inceptions to the present without language restrictions: MEDILINE, EMBASE, Cochrane Library, Web of Science, Cumulative Index to Nursing and Allied Health Literature, Allied and Complementary Medicine Database, Chinese Biomedical Literature Database, and China National Knowledge Infrastructure. We will consider all case-controlled studies investigating the impacts of UGFNAC diagnosis for patients with TN for inclusion. Two authors will independently carry out study selection, data collection, and methodological quality assessment. Quality Assessment of Diagnostic Accuracy Studies tool will be used for methodological quality evaluation. We will use RevMan V.5.3 and Stata V.12.0 software to perform statistical analysis.

**Results:**

We will apply sensitivity, specificity, positive likelihood ratio, negative likelihood ratio, and diagnostic odds ratio to judge the diagnostic accuracy of UGFNAC for TN.

**Conclusion:**

The results of this study will provide latest evidence for the diagnostic accuracy of UGFNAC for TN.

**Systematic review registration:**

PROSPERO CRD42019138805.

## 1 Introduction

Thyroid nodules (TN) are very common health disorders in clinical practice.^[1–3]^ It has been reported that such condition affects 19% to 68% healthy population.^[4,5]^ Of those, about 9% to 15% of TN is malignancy.^[5,6]^ Thus, it is very important to perform early diagnosis to exclude thyroid cancer and to enhance the survival rate of those patients.^[7]^ Ultrasound-guided fine needle aspiration cytology (UGFNAC) is a rapid, minimally invasive, and cost-effective diagnosis tool for patients with TN.^[8–19]^ Several previous studies have reported that UGFNAC has high diagnostic accuracy for patients with TN.^[8–19]^ However, there is still insufficient evidence-based medicine evidence to support it. Therefore, it is very necessary to conduct a study to systematically assess the diagnostic accuracy of UGFNAC for patients with TN.

## 2 Methods

### 2.1 Objective

The aim of the study is to evaluate the diagnostic accuracy of UGFNAC for patients with TN.

### 2.2 Study registration

This study has been registered on PROSPERO CRD42019138805. It reports are in accordance with the guideline of Preferred Reporting Items for Systematic Reviews and Meta-Analysis (PRISMA) Protocol statement.^[20]^

### 2.3 Eligibility criteria for study selection

#### 2.3.1 Types of studies

Case-controlled studies (CCSs) of TN that measure the value of diagnosis accuracy of UGFNAC will be included. No restrictions will be imposed to the basis of language of publications.

#### 2.3.2 Types of participants

Reports of participants with histological-proven TN will be considered for inclusion in this study.

#### 2.3.3 Type of index test

Index test: any forms of UGFNAC will be applied to diagnose patients with TN. However, studies will be excluded if they utilize the combinations of UGFNAC with other diagnostic test.

Reference test: patients with histological-proven TN will be used in the control group.

#### 2.3.4 Types of outcome measurements

The primary outcomes comprise of sensitivity and specificity. The secondary outcomes include positive likelihood ratio, negative likelihood ratio, and diagnostic odds ratio.

### 2.4 Data sources and search strategy

#### 2.4.1 Electronic searches

For this study we will search the following electronic databases from their inceptions to the present without language restrictions: MEDILINE, EMBASE, Cochrane Library, Web of Science, Cumulative Index to Nursing and Allied Health Literature, Allied and Complementary Medicine Database, Chinese Biomedical Literature Database, and China National Knowledge Infrastructure. All CCSs assessing the impacts of UGFNAC diagnosis for TN will be considered for inclusion. The exact search strategy sample for MEDILINE is presented in Table 1. Similar search strategy will be adapted to other electronic databases.

**Table 1:**
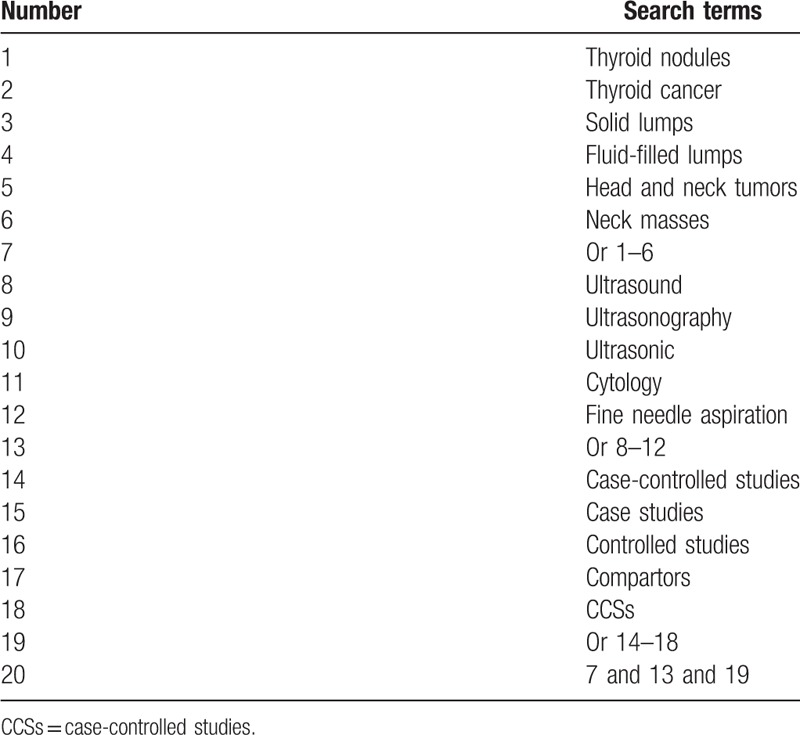
Search strategy for MEDILINE.

#### 2.4.2 Other resources

We will also search conference literatures, websites of clinical registry, and reference lists of relevant reviews to avoid losing more potential studies.

### 2.5 Data collection and analysis

#### 2.5.1 Selection of studies

The titles and abstracts of all searched literature records will be identified by 2 independent authors according to the pre-designed eligibility criteria. Any disagreements will be resolved by discussion with a third author. After irrelevant studies will be excluded, full copies of all remaining studies will be obtained. These studies will be further judged whether they meet the final inclusion criteria. We will provide explanation for each excluded study at different stages. The process of study selection is illustrated in the PRISMA flowchart.

#### 2.5.2 Data collection and management

Details of eligible studies will be extracted by 2 authors independently, and the results will be summarized using a data extraction form. A third author will help to solve the divisions between 2 authors.

The following information will be extracted for each eligible study:

- Study variables: such as title, author, year of publication, and location;
- Patient variables: such as age, sex, baseline characteristics, and inclusion and exclusion criteria;
- Study methods: such as details of randomization, concealment, blinding, and sample size;
- Intervention details: such as details of diagnostic methods, and comparators;
- Outcomes: such as number of true positives and negatives, false positives and negatives.

#### 2.5.3 Dealing with missing data

When an eligible study provided insufficient or missing information, we will approach primary authors for additional information. If we cannot get back that information, we will just analyze the available extracted data, and will also discuss its potential impacts in this study.

### 2.6 Methodological quality assessment for included studies

For this study, 2 authors will assess methodological quality for each eligible study using Quality Assessment of Diagnostic Accuracy Studies tool.^[21]^ It comprises of 4 aspects, and each aspect is further assessed and scored respectively. If there exists any divergences, a third author will be invited to help resolve those divergences through discussion.

### 2.7 Statistical analysis

In this study, we will utilize RevMan V.5.3 (Cochrane Community, London, UK) and Stata V.12.0 (StataCorp, College Station, TX) software to carry out statistical analysis. After data enter into the Stata V.12.0 software, we will plot the values of sensitivity, specificity, positive likelihood ratio, negative likelihood ratio, and diagnostic odds ratio from the diagnostic 2 × 2 tables of primary studies. We will calculate the descriptive statistics and 95% confidence intervals for each eligible study. In addition, we will also carry out descriptive forest plot and a summary receiver operating characteristic plot.

#### 2.7.1 Assessment of heterogeneity

*I*^2^ statistic test will be used for heterogeneity check among eligible studies. *I*^2^ ≤ 50% means low heterogeneity, while *I*^*2*^ > 50% means significant heterogeneity among the included studies.

#### 2.7.2 Data synthesis

If there is low heterogeneity (*I*^2^ ≤ 50%), we will pool the data, and will perform meta-analysis if it is possible. If there is significant heterogeneity (*I*^2^ > 50%), subgroup analysis will be performed, and meta-analysis will be operated according to the results of subgroup analysis. If there is still significant heterogeneity after subgroup analysis, we will not pool the data, as well as the meta-analysis will not be performed. Instead, we will apply bivariate random-effects regression approach to summarize the estimates of sensitivity and specificity.

#### 2.7.3 Subgroup analysis

We will carry out subgroup analysis to check any causes that may lead to significant heterogeneity based on the different study characteristics, as well as the patient characteristics.

#### 2.7.4 Sensitivity analysis

We will also perform sensitivity analysis by eliminating the low methodological quality studies.

#### 2.7.5 Reporting bias

We will perform funnel plots and relevant regression tests to identity if there are any publication biases.^[22]^

### 2.8 Ethics and dissemination

This study will not utilize personal data, thus, it does not require any ethic approval. Its results are expected to be published at peer-reviewed journals.

## 3 Discussion

UGFNAC is a safety, rapid, minimally invasive, and low cost for the diagnosis of TN. A numerous studies have reported that UGFNAC can effectively utilize as high accurate diagnosis tool for patients with TN. This study focuses on the diagnostic performance of different diagnosis that is presently applied in TN diagnosis and evaluation its potential role in decision-making for policy makers.

## Author contributions

**Conceptualization:** Jing Liu, Zhe Liu.

**Data curation:** Jing Wang, Jing Liu.

**Formal analysis:** Jing Wang, Jing Liu, Zhe Liu.

**Investigation:** Jing Wang, Zhe Liu.

**Methodology:** Jing Liu.

**Project administration:** Zhe Liu.

**Resources:** Jing Wang, Jing Liu.

**Software:** Jing Wang, Jing Liu.

**Supervision:** Zhe Liu.

**Validation:** Jing Wang, Jing Liu, Zhe Liu.

**Visualization:** Jing Wang, Jing Liu, Zhe Liu.

**Writing – original draft:** Jing Wang, Jing Liu, Zhe Liu.

**Writing – review & editing:** Jing Wang, Jing Liu, Zhe Liu.
